# The Control of Intemperance

**Published:** 1902-01-18

**Authors:** 


### The Hospital

A Journal of

The Medical Sciences and Hospital Administration.

Vol. XXXI.—No. 799.] Edited by Sir Henry Burdett, K.C.B., and Solomon C. Smith, M.D., M.R.C.P. [January 18, 1902.

### THE CONTROL OF INTEMPERANCE.

There are few more curious illustrations of the amount of wisdom with which the world is governed than those which are furnished by the sudden rise into prominence of questions which may, indeed, be of some actual importance, but which, for a longer or shorter time, are suffered to eclipse others which are at least equally worthy of attention. Four great causes of disease and of mortality in Great Britain are syphilis, intemperance, cancer, and tuberculosis; but while it has lately pleased the somewhat eccentric forces which govern public opinion to render the two last the subjects of unceasing chatter, amidst the turmoil of which quacks are reaping a rich harvest, the two former have been permitted of late almost to escape from the notice of sanitary reformers. Against syphilis, which works so much evil among the population, we have not only no defence, or no attempt at any; but such as there once was has been taken away. The question of intemperance, however, may perhaps be considered to rest upon a somewhat different basis, and to lend itself more readily to control by reasonable and practicable means.

The current number of the *International Journal of Ethics* contains an exceedingly suggestive paper on this subject by Professor Hyslop, of the Columbia University of New York, who, speaking from the large experience of the United States, declares, without even thinking it necessary to demonstrate, that every kind of prohibitory measure with regard to the supply of drink has so far been an absolute and unredeemed failure. He assumes this as an incontestable position; and, as our space will not allow us to quote the evidence on which it rests, we may point out that much of this may be found in the recently published work of Dr. Archdall Reid, entitled "Alcoholism and Heredity." Professor Hyslop declares that it is time for temperance reformers to cease from thinking of the "supply" of alcohol, and to confine their efforts to endeavours to limit the "demand." As long as the demand exists, the supply will follow it; and all stringent laws will be evaded, just as a prohibitory tariff is a direct incentive to smuggling. The remedy for intemperance which he proposes is founded upon the principle of public guardianship; that is to say, upon the deprivation of a person convicted of drunkenness of the power to receive and deal with money. He relates some very interesting experiences of a gentleman named Swan, of Norwich, Connecticut, who set himself to deal with the local drunkenness, and who obtained the co-operation of the local judiciary. It seems that the laws of the State permit a sentence of six months' imprisonment for drunkenness, but that the customary term was from 20 to 30 days. Mr. Swan persuaded the magistracy always to pass the extreme sentence, and he then went to the culprit and offered to get him off on condition that he would sign a paper authorising him (Mr. Swan) to receive and lay out his wages for the period. This being done in legal form, Mr. Swan appealed to the magistrate to suspend the operation of the sentence for 60 days, and the appeal was by pre-arrangement consented to. The culprit then went back to his work, and Mr. Swan received his money, and issued tickets to tradesmen authorising them to supply specified necessaries in specified quantities for the maintenance of the family. The culprit received a monthly statement of the sums so expended and of the amount standing to his credit, and at the end of 60 days appeared in court, to receive, if his conduct had been satisfactory, a further suspension of his sentence for the same period. At the end of the third term of 60 days, if all went well, he was released from control. In the intervening period he would often have gained greatly in health comfort, and efficiency; he would have been well fed; and the comforts of his home would have been materially increased; as Mr. Swan did not hesitate to issue tickets for the purchase of furniture or clothing. The results of the system have been sufficient to lead some to think that it might with advantage be embodied in an Act of the State Legislature, and that the necessary "guardianship" might be entrusted to some public authority, either existing or established for the purpose. Cases would no doubt occur in which the man under guardianship would endeavour to sell or pawn his goods, or would be treated to drink by his fellow-workmen, and these should be met by the sharp punishment of all concerned. The city of Norwich contains a population of about 30,000, and it is obvious that a place of this size affords facilities for supervision which would not exist in a great centre of industry; but still Mr. Swan's experience is distinctly valuable, and is at all events better than the truly British method of passing of an Act for the confinement of inebriates without making any provision of places in which they may be confined.

